# Cytokines in the genesis and treatment of cancer

**DOI:** 10.1038/sj.bjc.6604050

**Published:** 2007-11-27

**Authors:** A G Dalgleish

**Affiliations:** 1 St Georges Hospital, University of London, UK

Cytokines are the soluble messengers that immune cells produce to either attack an invading organism or to talk to other types of immune cells. They form part of the complex pattern of the immune response, which can assist the development of cancer as well as to eliminate it. In the foreword of this book, Glenn Dranoff draws attention to the fact that the cytokines which are involved in the chronic inflammatory pathways that are often associated with cancer development (citing Hal Dvorak's concept of cancer as a wound that does not heal) may, while trying to effect the tissue repair, perhaps unintentionally promote, tumour cell growth, attenuate apoptosis and facilitate angiogenesis invasion and metastases. Whereas cytokines are actively involved in this process they have also been singled out for use as possible treatments. Interferon and interleukin-2 are well-known examples of cytokines that have been approved for cancer treatments and one of these cytokines known as tumour necrosis factor-*α* (TNF*α*) exemplifies the paradox that it was identified as a ‘tumour necrosis factor’ in mice (and is still given to patients to kill tumours, such as in limb perfusion) for melanoma and sarcoma, yet when chronically produced by nearly all the cells of the immune system it can cause the proliferation, invasion and metastases of tumour cells, hence inhibiting it is also a rational target.

The book sets out to examine the role of cytokines in the development of cancer and the first section focuses specifically on chronic infectious diseases and the cytokines they produce and the formation of cancer with specific chapters on *Helicobacter pylori* and gastric cancer, HTLV-1 and the adult T-cell leukaemic lymphoma syndrome, the herpes viruses and the association of EBV with diffusely different tumours from Burkett's lymphoma to nasopharyngeal carcinoma. The fact that this is a common virus and the tumours relatively uncommon means that there is a very complicated interaction with the environment and the immune system and this group of viruses are of particular interest as they encode cytokine homologues, IL-10 in the case of EBV and interleukin-6 and the viral interferon regulatory factors in the case of human herpes virus type 8 (which causes Kaposi's sarcoma). The book then goes on to explore the possible similarities between the chronic infectious state and non-infectious, pre-cancer conditions associated with cancers and the role of cytokines, and the first one being the paradox of the TNF*α* is reviewed in detail. Drugs targeting TNF*α* are already making a big clinical impact, such as the thalidomide family with analogues such as lenalidomide already being approved for use in myelodysplastic syndrome and myeloma. Both thalidomide and lenalidomide were selected purely for the ability to inhibit the TNF pathway but it is now accepted that they are active in a number of pathways, including antiangiogenic and anti-inflammatory ones. The chapter describes the development of infliximab, a monoclonal antibody, which is being trialled as an anticancer agent following its very successful use in treating debilitating inflammatory disorders, such as rheumatoid arthritis and Crohn's disease.

The link between infectious agents, cytokines and cancer, as well as the paradoxical actions of certain cytokines makes an excellent introduction to what is a very comprehensive reference book. There are major chapters on transforming growth factor-*β*, the interleukin-1 family, the interleukin-4/13 family, interleukin-6 and interleukin-10. All these chapters explore in depth the relationship of these cytokines with cancer. In the case of interleukin-6, there is a particular focus on Castleman's disease. Then follows chapters on the role of cytokines in multiple myeloma and the therapeutic implications followed by two chapters on experimental cytokine models and the genesis of cancer as well as the implications for cancer prevention. The third section of the book focuses on cytokines and the tumour stroma and their role in angiogenesis and tumour progression, with one chapter focussing on the biology of cancer, cachexia and the role of TNF *α* in it. The fourth and final section of the book is devoted to cytokines and the treatment of cancer with the cytokines not previously reviewed in depth, currently in clinical use (or are in the development phase), such as interleukin-2, the interferons and interleukin-12, which are covered in separate chapters. Indeed, this last section should be compulsive reading for all cancer physicians. In addition to the good in-depth reviews of the clinically available cytokines already mentioned, there are some very useful, practical chapters and in particular, the one on combination cytokine therapy. An in-depth knowledge of the role of all the various cytokines strongly suggests that the combinations should be synergistic and this field is reviewed in depth. Indeed, it is surprising how many combinations have already been tried and reported in the clinic, not only are there many doublet combinations but also triplet ones involving the interferons, IL-2 and GMCSF. There is far too much information to précis here but the field is plagued with doubts about the correct doses to use and the optimal sequences to give the cytokines. Logical sequences give the desired predicted result such as when IL-2 and IL-12 are given, there is enhanced γ interferon and NK activity. However, although dramatic clinical responses are seen, one cannot avoid the conclusion that these responses occur no more frequently than would be expected with either cytokine alone. Nevertheless, it is possible that the dosing and sequencing of these combinations are crucial and that some of the doses used, with significant side effects when they are too high, may prevent the ability of any potential synergy to occur. Indeed, it is hard to believe that very high doses of single cytokines, causing significant systemic toxicity can act independently alone but are probably inducing a cytokine storm which could probably be induced by injecting a highly infectious, dangerous agent and this of course brings Cooley's toxins to mind!

The final chapters deal with the potential of using fusion proteins to target cytokine administration as well as the use of cytokine-transfected cells as vaccines. The use of cytokines to boost immune responses to non-transfected vaccines is a minor omission here. There is a review of cytokines used in support of care, which most clinicians will be familiar with, such as erythropoietin, GCSF, etc., and a separate chapter exploring the potential of anticytokine therapy based on inhibiting interleukin-1 and TNF. Interestingly, they do not mention IL-6, which is currently being developed as a target by Centocor for a number of conditions associated with a high IL-6 level.

In conclusion, this is a fascinating, comprehensive book on cytokines in cancer, both as causative agents and potential treatments. I was interested to hear a recent postgraduate entry student complain to me that the most disappointing thing about her course was the virtual absence of immunology and immunotherapy in it when it was clearly going to be the treatment of the future! As her experience is not unique in the UK, I can only recommend that in order to help this deficit that this book should be one of the several in this field that should be compulsive reading for clinicians, students and their non-clinical teachers alike!

